# Signal quality evaluation of an in-ear EEG device in comparison to a conventional cap system

**DOI:** 10.3389/fnins.2024.1441897

**Published:** 2024-09-10

**Authors:** Hanane Moumane, Jérémy Pazuelo, Mérie Nassar, Jose Yesith Juez, Mario Valderrama, Michel Le Van Quyen

**Affiliations:** ^1^Laboratoire d’Imagerie Biomédicale (LIB), Inserm U1146, Sorbonne Université, CNRS UMR7371, 15 rue de l’Ecole de Médecine, Paris, France; ^2^Department of Biomedical Engineering, Universidad de Los Andes, Bogotá, Colombia

**Keywords:** EEG, in-ear device, signal quality, scalp EEG, wearable technology

## Abstract

**Introduction:**

Wearable in-ear electroencephalographic (EEG) devices hold significant promise for integrating brain monitoring technologies into real-life applications. However, despite the introduction of various in-ear EEG systems, there remains a necessity for validating these technologies against gold-standard, clinical-grade devices. This study aims to evaluate the signal quality of a newly developed mobile in-ear EEG device compared to a standard scalp EEG system among healthy volunteers during wakefulness and sleep.

**Methods:**

The study evaluated an in-ear EEG device equipped with dry electrodes in a laboratory setting, recording a single bipolar EEG channel using a cross-ear electrode configuration. Thirty healthy participants were recorded simultaneously using the in-ear EEG device and a conventional EEG cap system with 64 wet electrodes. Based on two recording protocols, one during a resting state condition involving alternating eye opening and closure with a low degree of artifact contamination and another consisting of a daytime nap, several quality measures were used for a quantitative comparison including root mean square (RMS) analysis, artifact quantification, similarities of relative spectral power (RSP), signal-to-noise ratio (SNR) based on alpha peak criteria, and cross-signal correlations of alpha activity during eyes-closed conditions and sleep activities. The statistical significance of our results was assessed through nonparametric permutation tests with False Discovery Rate (FDR) control.

**Results:**

During the resting state, in-ear and scalp EEG signals exhibited similar fluctuations, characterized by comparable RMS values. However, intermittent signal alterations were noticed in the in-ear recordings during nap sessions, attributed to movements of the head and facial muscles. Spectral analysis indicated similar patterns between in-ear and scalp EEG, showing prominent peaks in the alpha range (8–12 Hz) during rest and in the low-frequency range during naps (particularly in the theta range of 4–7 Hz). Analysis of alpha wave characteristics during eye closures revealed smaller alpha wave amplitudes and slightly lower signal-to-noise ratio (SNR) values in the in-ear EEG compared to scalp EEG. In around 80% of cases, cross-correlation analysis between in-ear and scalp signals, using a contralateral bipolar montage of 64 scalp electrodes, revealed significant correlations with scalp EEG (*p* < 0.01), particularly evident in the FT11-FT12 and T7-T8 electrode derivations.

**Conclusion:**

Our findings support the feasibility of using in-ear EEG devices with dry-contact electrodes for brain activity monitoring, compared to a standard scalp EEG, notably for wakefulness and sleep uses. Although marginal signal degradation is associated with head and facial muscle contractions, the in-ear device offers promising applications for long-term EEG recordings, particularly in scenarios requiring enhanced comfort and user-friendliness.

## 1 Introduction

Wearable devices are increasingly present in healthcare as tools for biomedical research or clinical applications. Their growing development has been accelerated by recent technological progress, which combines skin-attachable physiological monitoring sensors with compact and high-performance recording components. One type of wearable device that has gained attention is those worn in or around the ear, known as “earables. Positioned uniquely on the human head, these devices offer a specialized location for sensing various physiological parameters, including face, eye, head movements, body sounds, heart rate, blood oxygen saturation, or respiration (Röddiger et al., 2022). This technology takes advantage of the anatomical characteristics of the ear that offer a convenient dock to host the required electronics needed to fit a wearable device. Most importantly, they are discrete and unobtrusive as they are similar to audio devices people commonly use, such as earbuds or earplugs.

Due to its proximity to the brain, the external ear offers an interesting location for monitoring brain activity. Specifically, various types of wearable devices for recording brain electrical activity, known as electroencephalogram (EEG), have been developed (Kaongoen et al., 2023). These devices utilize electrodes placed in different areas within the outer ear, predominantly in the ear canal (Looney et al., 2011; Goverdovsky et al., 2017). The interest in this type of technology comes from the use of small electrodes (each with an area of approximately 9 mm^2^) that are easy to wear, significantly reducing setup time compared to traditional EEG systems, which require the placement of multiple electrodes on the scalp by trained personnel. Additionally, the tight fit of an earpiece in the ear canal applies pressure on the electrodes, ensuring stable electrode positions and partially reducing motion artifacts that commonly degrade signal quality in conventional EEG recordings (Mikkelsen et al., 2015). The initial development of the in-ear EEG device by Looney et al. (2011) marked a significant breakthrough in this wearable monitoring technology. Since its introduction, this technology has been rigorously tested and proven effective in over 90 peer-reviewed studies (Juez et al., 2024), highlighting improvements in materials, system design for everyday use, and the reliability of signal quality (Correia et al., 2024). Additionally, innovations have included sensor designs such as custom 3D molded impressions tailored to individual ear shapes (Valentin et al., 2021) and the use of memory foam for better comfort and fit (Goverdovsky et al., 2016). Tabar et al. (2023) further advanced the technology by creating a universal earplug made from soft silicone, noted for its high-quality signal and suitability for extended monitoring. This ear-EEG technology has found diverse applications, ranging from monitoring emotional and stress levels (Athavipach et al., 2019; Lee et al., 2020) to more specialized uses such as hearing tests (Christensen et al., 2018), securing personal authentication systems (Merrill et al., 2019), and detecting drowsiness in drivers (Hong et al., 2018). In medical contexts, these devices have been especially valuable. They are used for sleep analysis, performing comparably to the gold-standard polysomnography to assess sleep stages accurately (Mikkelsen et al., 2017a). The technology is also gaining recognition for its potential to monitor epileptic seizures, allowing continuous, real-time observation outside hospital environments and improving diagnostic and follow-up processes (Zibrandtsen et al., 2017; Joyner et al., 2024). Recent studies have shown its effectiveness for long-term EEG monitoring in patients with Alzheimer disease (Musaeus et al., 2023a) and Lewy body dementia (Musaeus et al., 2023b), both conditions that significantly increase the risk of epileptic disorders. Overall, in-ear EEG technology holds the potential for developing new monitoring procedures for various clinical conditions.

Specifically, numerous studies have demonstrated that EEG signals captured from the ear canal closely resemble those obtained from scalp electrodes located near the ear, whether during cognitive activities (Kidmose et al., 2012) or sleep (Zibrandtsen et al., 2016). In particular, (Looney et al., 2011) proved high coherence between an in-ear EEG electrode and the standard scalp T7-M1 electrode (ipsilateral mastoid reference) from the international 10–20 placement system, reflecting the shared activity between the temporal lobe and in-ear locations. However, the signal recorded inside the ear typically has a lower amplitude than scalp EEG (Mikkelsen et al., 2015). This is likely due to the greater distance from the brain’s generating sources to the recording sites inside the ear and the electrical and geometric properties of the electrodes used for recording. In general, recording high-quality bioelectrical signals from electrodes placed within the ear relies critically on the electrode-skin interface. Several ear-EEG studies have been performed with wet electrodes, in which conductive gel or hydrogel was applied between the electrodes and the skin (Mikkelsen et al., 2015). Nevertheless, dry-contact ear-EEG electrodes would increase the comfort and user-friendliness of these devices (Mikkelsen et al., 2019). However, these electrodes are not without constraints, notably exhibiting significantly higher impedance at the electrode-skin interface (200–1,000 kΩ vs. 10–20 kΩ) (Chi et al., 2010). Therefore, a lower signal quality is expected, and data quality loss should be evaluated by concurrent recording of the proposed in-ear EEG system alongside conventional gel electrodes positioned on the scalp.

In this study, we conducted a systematic signal evaluation of a mobile in-ear EEG device developed by Naox Technologies. This device comprises non-invasive dry electrodes paired with a miniaturized electronic system that captures EEG signals from the subject’s ear canals. Our primary objective was to validate the EEG signal of the in-ear system against a research-grade EEG system equipped with a 64-electrode cap in laboratory conditions, using a sample of healthy volunteers during both wakefulness and sleep periods. Our secondary objective was to assess the signal quality of the in-ear system over several hours of continuous recording compared to scalp electrodes, with a particular focus on electrodes located at temporal positions T7-T8.

## 2 Methods

### 2.1 The in-ear EEG device

**Figure 1:**
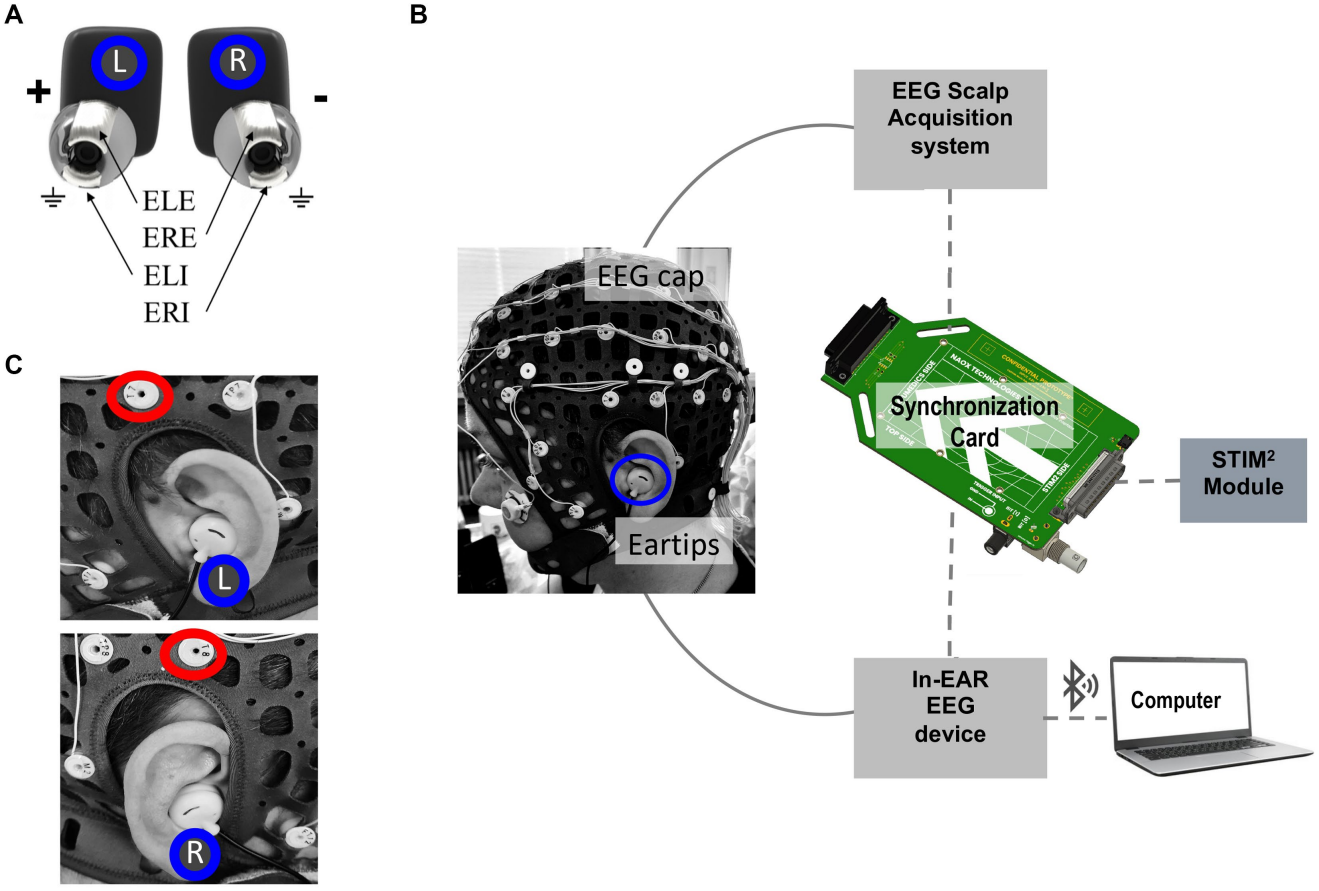
Experimental setup for EEG recording used in this study. **(A)** Close-up view of the in-ear EEG device developed by Naox Technologies, with electrodes inserted in positions ELE, ERE, ELI, and ERI, corresponding to two contact points inside each ear canal. **(B)** Experimental setup for simultaneous scalp and in-ear EEG acquisition. The EEG cap with 64 scalp electrodes and eartips for in-ear EEG recording. The EEG signals from the scalp and in-ear devices are synchronized via a synchronization card that receives triggers from the STIM^2^ device. The in-ear EEG signals are transmitted to a computer via Bluetooth. **(C)** Detailed images showing the placement of the in-ear EEG device in the left (L) and right (R) ears. Red circles highlight the positioning of T7 and T8 scalp electrodes used for comparison.

The in-ear EEG device, created by Naox Technologies (Figure 1A), follows established scientific recommendations. The electrodes, made from silicon and coated with conductive silver ink for optimal biocompatibility and conductivity, have surface areas between 8 mm^2^ and 11 mm^2^. The device features four electrodes, designated as ERE, ERI, ELE, and ELI, corresponding to two contact points within each ear canal (left and right). Employing a cross-ear electrode configuration (Left Ear Superior - Right Ear Superior, ELE-ERE), the system functions as a single bipolar EEG channel, detecting voltage differences between electrodes in opposite ear canals. The ground (GND) connection is achieved by linking the lower electrodes (ELI and ERI) within both ear canals.

An electronic board amplifies the EEG signal and converts it to a digital format with a sampling rate of 250 Hz and 24-bit resolution. The data is transmitted in real-time via Bluetooth Low Energy 2.4GHz (BLE) to a laptop. The device’s battery supports up to 10 h of continuous recording, allowing for prolonged monitoring sessions. Weighing approximately 20 grams, it provides a lightweight and non-intrusive user experience. Additionally, the system complies with electrical safety standards, including IEC 60601–1, IEC 60601–1-2, IEC 80601–2-26, IEC 60601–1-11, and IEC 62133, ensuring its safety and reliability across various applications.

**Figure 2:**
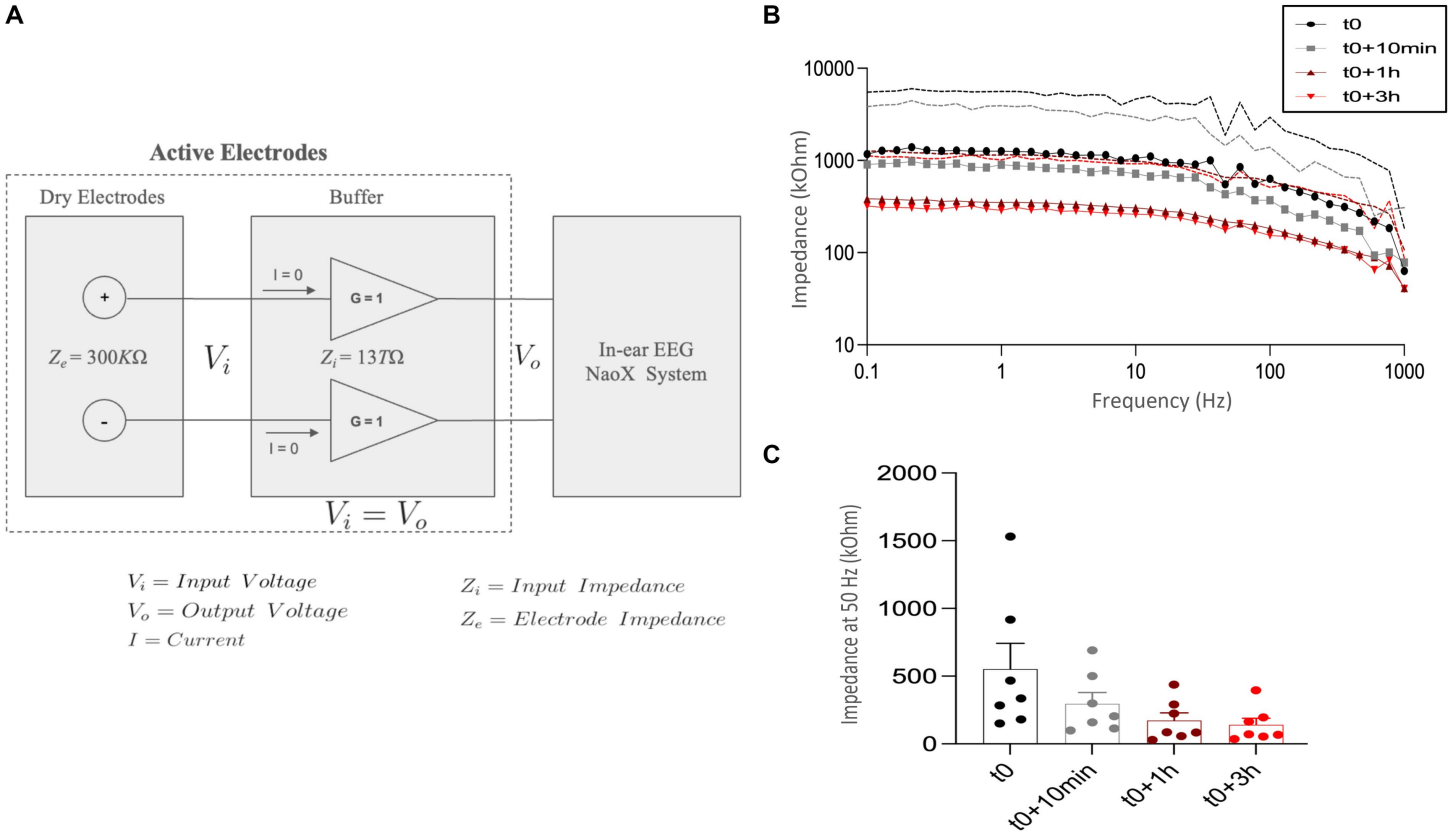
In-ear EEG impedance measurements over time. **(A)** Architecture of the in-ear EEG system configuration. Dry electrodes with an impedance (Ze) of 300 kΩ are connected to active electrodes with a high input impedance (Zi) of 13 TΩ and unity gain (G = 1). This setup ensures the input voltage (Vi) equals the output voltage (Vo), minimizes current flow (I = 0), and reduces noise, preserving signal quality for accurate EEG signal transmission to the Naox system. **(B)** Impedance spectra measured at different time points: at the initial time (t0, black circles), 10 min after the initial time (t0 + 10 min, gray squares), 1 h after the initial time (t0 + 1 h, red triangles), and 3 h after the initial time (t0 + 3 h, red diamonds). The impedance is plotted against frequency (0.1 Hz to 1,000 Hz) on a logarithmic scale. Dashed lines represent the standard deviation (SD). **(C)** Impedance at 50 Hz measured at the same time points: t0, t0 + 10 min, t0 + 1 h, and t0 + 3 h. The y-axis represents impedance in kilo-ohms (kΩ). Data is presented as mean ± standard deviation (SD) with individual data points for the different participants (*N* = 7) plotted.

A notable challenge when using dry electrodes, such as those in the in-ear Naox device, is their inherently higher impedance (Ze = 300 kΩ, see Section 4.2), which tends to increase noise levels. To mitigate this issue, active electrodes with a high input impedance (Zi = 13 TΩ) were integrated into the earplugs. This design ensures that the input voltage (Vi) equals the output voltage (Vo), as depicted in Figure 2A. Choosing a buffer with such a high input impedance, much greater than the electrode-skin impedance of 300 kΩ, helps minimize noise due to common mode and external interference as a consequence of impedance adaptation. By aligning the high input impedance of the active electrodes with the impedance at the electrode-skin interface, we effectively reduce noise and maintain the integrity of the EEG signals.

### 2.2 Scalp EEG acquisition system

The general benchmarking framework in this study aims to compare EEG signals captured by the in-ear wearable device and those recorded using gold-standard EEG monitoring equipment featuring wet scalp electrodes. In our study, we utilized a 64-channel Compumedics NEUVO amplifier for scalp EEG signal acquisition (Figures 1B,C), with a sampling rate of 2000 Hz, and Curry 8 software for data acquisition. The scalp electrodes were positioned according to the 10–10 international system. Skin preparation involved the application of a conductive gel (Ten20) to enhance electrode-skin conductivity. Additionally, for the sleep protocols, we included two electrooculogram electrodes and one chin electrode for sleep scoring. Before all experiments, we checked scalp EEG signals to maintain optimal impedances (< 5 kΩ) and performed a visual inspection of signal quality to identify and discard any instances of failure in electrode-skin contact. These aspects were evaluated based on the best practice guidelines and recommendations for EEG studies (Keil et al., 2014).

### 2.3 Synchronization between the in-ear and scalp systems

Achieving precise temporal synchronization (around 1 ms) between the scalp and in-ear EEG devices was crucial for ensuring accurate signal comparison in our analysis. To address this challenge, we utilized the STIM2 system from Compumedics Neuroscan, known for its high-precision stimulus presentation. According to the experimental protocol, the STIM2 system delivered triggers simultaneously to the Compumedics (scalp) and Naox (in-ear) systems. To facilitate this synchronization, we developed an electronic synchronization card connecting the STIM2 system to Compumedics and Naox devices via a jack cable (Figure 1B). This setup allowed for precise alignment of triggers, ensuring synchronization below the millisecond threshold. After data acquisition, the recorded signals from both devices underwent further alignment using a custom software routine. This offline process involved detecting trigger markers through peak detection from the absolute values of the trigger signals. In a single case (subject 21), synchronization was not possible due to missing triggers recorded by the in-ear device during the alpha test.

### 2.4 Experimental protocols and procedures

We enrolled 30 healthy controls (20 females and 10 males; Age: 26.9 ± 6.5 years, range: 20–46 years). The selected subjects were not treated with any medications, had no history of substance abuse or dependence, and did not have a neurological or psychiatric illness, head trauma/stroke, sleep or hearing disorders (pathologies affecting the inner and middle ear). All participants provided written informed consent before participation, and the ethical committee of Sorbonne University approved the study. The data acquisition experiment was conducted at the Laboratoire d’Imagerie Biomédicale (LIB), from September 2023 to February 2024.

Each participant first attended an earbud fitting session before the actual recordings. During this session, the operator chose earpieces that best fit the participant’s ears (3 sizes: S, M, and L) and with acceptable signal quality characterized by visual inspection of the in-ear EEG on the acquisition laptop. Altogether, the majority of the participants (57%) used the medium-sized “M” earpieces, whereas the small-sized “S” ones were better suited for 43% of the subjects. Before inserting the earpieces, the ear canals were cleaned with cotton swabs (Q-tips). For the actual recording session, the operator first placed a 64-EEG electrode cap with conductive gel on the scalp. Subsequently, the participant placed the EEG earbuds on each ear without conductive gel (dry-contact electrodes).

Dual in-ear and scalp EEG recordings started around 2 pm and continued throughout the afternoon (until 5 pm). Two experimental sessions were recorded: In the first protocol (the “Alpha test”), participants alternated between opening and closing their eyes for 30 s in response to an auditory cue that signaled a change in condition. A low-pitched sound indicates the eyes-closed condition, and a high-pitched sound indicates the eyes-open condition. The sequence always began with the eyes-closed condition. Each session comprised 10 trials, split evenly between eyes closed and eyes open, totaling a duration of 5 min per subject. Across all participants, the combined duration of the recordings reached 145 min. All participants sat comfortably in a quiet room. As in standard EEG protocols, participants were instructed to stay still and concentrate on a cross in front of them to minimize significant movements of the head and facial muscles during recordings, thereby reducing EEG artifacts, especially those related to face movements. During the second condition (the “nap test”), the participants were invited to have an afternoon nap of around 1 h (a total of 26.5 h was recorded among all subjects). During the measurements, the subjects were placed in a relaxed supine position and encouraged to relax or, if possible, to sleep. No recommendations were provided concerning head or body movements. After the recording sessions, each participant completed a poststudy survey to evaluate the comfort and usability of the device.

### 2.5 In-ear EEG impedance measurements

Electrode-skin impedance is a critical factor for assessing the quality of EEG signals, influenced by elements such as electrode material, design, and skin characteristics. Generally, dry electrodes exhibit higher impedance compared to wet electrodes; however, factors like sweating can lower this impedance, improving signal quality (Chi et al., 2010). Specifically, in-ear dry electrodes show a reduction in impedance over time, eventually stabilizing to levels similar to those of wet electrodes (Xu et al., 2023). To investigate the impedance changes in the ear canal using the in-ear electrodes from this study, we adhered to established protocols from previous research (Kappel and Kidmose, 2015; Kappel et al., 2019; Mandekar et al., 2022). From the enrolled participants, we randomly selected seven subjects before the experimental sessions. Their ear canals were first cleaned with ear swabs prior to electrode insertion and measurement. We employed a PalmSens4 impedance analyzer (PalmSens BV, The Netherlands), connecting ear tip electrodes to the working (ELE) and reference (ERE) electrodes (Shin et al., 2022). Impedance spectra were recorded from 0.1 to 1,000 Hz at four intervals: immediately after insertion (t0), after 10 min (t0 + 10 min), after 1 h (t0 + 1 h), and after 3 h (t0 + 3 h). Measurements were conducted in a contralateral configuration for both ears simultaneously, with each measurement interval lasting approximately 2 min. We calculated the mean, standard deviation (SD), and standard error of the mean (SEM) for the impedance spectra and obtained resistor and capacitor values using the PalmSens4 fit tool.

## 3 Signal processing

### 3.1 Root mean square values of the in-ear and scalp EEG signals

Root mean square (RMS) metrics are commonly used in studies comparing different types of EEG equipment to evaluate signal quality (Shin et al., 2022; Tabar et al., 2023; Erickson et al., 2024). Following these studies, we first preprocessed raw signals by applying a finite impulse response (FIR) bandpass filter (0.3–35 Hz) to remove slow trends while retaining as much EEG information as possible. The motivation behind this filtering approach was to capture a broad range of EEG frequencies pertinent to various wake and sleep states, including slow-wave activity (0.5–4 Hz) and faster rhythms up to 35 Hz. Subsequently, we calculated the average RMS values for epochs of 10 s (non-overlapping). The data underwent artifact rejection criteria, where we considered only windows that did not exceed the threshold of −100 μV to +100 μV (for at least 10% of the time) for our analysis. A window duration of 10 s was commonly adopted in artifact detection (Lopes et al., 2023) because it balances capturing sufficient data for robust statistical analysis while minimizing the effects of transient artifacts. In contrast, longer durations like 30-s windows are more prone to artifacts that could skew the analysis. Utilizing shorter epochs helps mitigate these issues, thereby enhancing the reliability and accuracy of the results.

### 3.2 Alpha peak criteria for the eye closed condition

Our analysis focused specifically on the 5 trials from the “Alpha test” during which participants kept their eyes closed for 30 s. To create time-frequency spectrograms for each subject’s signals, we first implemented a wavelet decomposition using the continuous Gabor Wavelet described by Morlet et al. (1982). Next, we characterized the increase in the power of the alpha band (8–12 Hz) through the signal-to-noise ratio (SNR) within the alpha frequency range that we calculated using the formula established by Tautan (2014):


$$SNR\left(\mathrm{alpha}\right)=\frac{\mathrm{mean}\left(\mathrm{Power}\;8-12\;\mathrm{Hz}\right)}{\mathrm{mean}\left(\mathrm{Power}\;5-35\;\mathrm{Hz}\;\mathrm{without}\;7-13\;\mathrm{Hz}\right)}$$

We adopted a criterion for a “clear peak” as an SNR value exceeding 1.5, corresponding to a 3.52 dB amplitude difference relative to surrounding noise.

### 3.3 Correlations between in-ear and scalp EEG

We estimated the Pearson correlation coefficient during the eyes-closed condition to measure the similarity of the in-ear signals with the scalp EEG in the alpha frequency band (8–12 Hz). In particular, using a contralateral bipolar montage, we calculated the normalized cross-correlations between the in-ear signals and each of the 64 scalp electrodes. For this, we used MATLAB’s “xcorr” function, with a lag of 0.1 s, after passband filtering within the alpha range. The lag was implemented to mitigate a possible drift occurring between the clocks of the two independent EEG systems, which leads to a slight misalignment between the signals. Also, to ensure uniformity, we harmonized the sampling frequency of the scalp signal with that of the in-ear EEG, setting both at 250 Hz. Similarly, during the “Nap tests,” we quantified the correlation coefficients between in-ear and scalp EEG signals for each sleep stage (Wake, N1, N2, N3, REM). Here, we computed the correlation between the in-ear signal and one bipolar scalp EEG channel (T7-T8) using 10-s sliding windows and broadband filtering within the 0.3–35 Hz range. These correlations were interpreted based on conventional criteria: poor (<0.02), fair (0.2–0.4), moderate (0.4–0.6), substantial (0.6–0.8), and almost perfect agreement (>0.8) (Landis and Koch, 1977).

### 3.4 Sleep scoring

Sleep scoring was conducted using scalp EEG and EOG signals through USleep (Model U-sleep-FT V2.0), an online platform specifically designed for automated sleep staging. USleep is a publicly available, ready-to-use deep neural network for resilient sleep staging inspired by the popular U-Net architecture for image segmentation. This model is based on a deep neural network with multiple convolutional layers of varying kernel sizes and strides to capture both short-term and long-term dependencies in the EEG and EOG signals and to extract features from the input signals. These layers are followed by max-pooling layers, which reduce the spatial dimensions and computational complexity while preserving essential features. After the convolutional and pooling layers, the network includes fully connected layers that perform the final classification into different sleep stages. From its previous version, the model was improved by fine-tuning using a larger and corrected training dataset, which enhanced its performance across different patient groups. The USleep-FT V2.0 model requires two input channels, which can be a combination of EEG and EOG signals. It was trained on a comprehensive polysomnography (PSG) recordings dataset, totaling 25,696 records from 16 clinical cohorts. The model’s effectiveness was thoroughly tested on 8 separate clinical cohorts with 346 PSG records. The F1 scores obtained by the model were on par with the top clinical experts. Additionally, a detailed cross-validation process was used during training and testing to ensure the model’s robustness and ability to generalize to new data (Perslev et al., 2021). As recommended by the American Academy of Sleep Medicine, the scalp derivations used for automatic sleep scoring included the electrode derivations ‘F4-M1’, ‘C4-M1’, and ‘O2-M1’, along with EOG channels (HEOG and VEOG) recorded during nap sessions (Berry et al., 2020). Following additional recommendations, the scalp signals were initially preprocessed and filtered within a frequency range of 0.3 to 35 Hz. Sleep stages were successfully scored in standard 30-s epochs for 22 subjects. It’s important to note that the model employed in our study was not specifically adjusted or fine-tuned with the EEG data we used. This means that we applied the model with its predefined configurations without optimizing it for the unique characteristics or specific variations of our EEG data. Also, it should be noted that we performed sleep scoring solely using scalp EEG data, and the resulting hypnograms were then utilized to analyze the in-ear EEG data. These in-ear recordings were collected simultaneously and synchronized with the scalp EEG data. However, in 7 other subjects, interruptions in the in-ear signal recordings made synchronization difficult, resulting in the rejection of the corresponding data for these cases. In total, Wake was consistently observed in all 22 subjects. The N1 phase was observed in 20 out of 22 subjects (91%), the N2 phase in 19 out of 22 recordings (86%), the N3 phase in 7 out of 22 recordings (32%), and the REM phase in 4 out of 22 recordings (18%). After sleep scoring, we quantified the average relative spectral power (RSP) of the in-ear and scalp EEG during each stage (Wake, N1, N2, N3, REM). To do this, we performed a Fast Fourier Transform (FFT) analysis for each subject using sliding windows of 10 s. With these relative power values of a given frequency, we then defined the ratio of the sum PSD in this frequency to the sum PSD in a wide frequency range (0.3 Hz, 35 Hz). Finally, we statistically compared in-ear and scalp EEG in the standard frequency bands: delta: 0.3–4 Hz, theta: 4–8 Hz, alpha: 8–12 Hz, beta1: 12–18 Hz, and beta2: 18–35 Hz. Statistical tests could not be performed for the N3 and REM phases due to an insufficient number of subjects (7 and 4, respectively) presenting these two sleep stages.

### 3.5 Statistical analysis

To determine the statistical significance of the linear correlation coefficients obtained from in-ear and scalp EEG signals, we conducted a Pitman nonparametric permutation test following established methodologies detailed in prior literature. Nonparametric statistical testing, commonly employed in neuroimaging studies (Nichols and Holmes, 2002), offers the advantage of not relying on population parameters or knowledge of the sampled population (Daniel and Cross, 2018). Our approach closely followed procedures outlined in previous studies (Theiler et al., 1992; Maris and Oostenveld, 2007; Haaga and Datseris, 2022), involving the creation of surrogates by randomly shuffling short-time block intervals or trials in the time domain. Specifically, we fixed one signal and randomly shuffled all corresponding 10-s intervals from the second signal. Subsequently, we computed the Pearson correlation coefficient between the fixed signal and the shuffled signal intervals. As recommended by Butar and Park (2008), this procedure was repeated 1,600 times to generate a distribution of correlation coefficient values from permutations to achieve robust statistical inference from permutation tests. A two-sided *p*-value was calculated by comparing the absolute values of correlation coefficients from shuffled permutations to the true absolute value obtained from the original data series. In cases requiring multiple comparisons, such as when analyzing various EEG channels or different time intervals, we controlled the False Discovery Rate (FDR) using the Benjamini and Hochberg (1995) method.

We adopted a similar permutation-based procedure for all statistical comparisons involving amplitude and power measures between scalp and in-ear signals. In this instance, we permuted all values from the original distributions of measures, comparing them to obtain the difference between means for each permutation. We then estimated the two-sided p-value as the proportion of absolute values of mean differences from permutations that exceeded the true absolute value of the mean difference calculated from the original distribution of measures, with a statistical significance level defined at *p* < 0.01. We then controlled the False Discovery Rate (FDR) using the Benjamini-Hochberg method.

## 4 Results

### 4.1 Comfort and technical issues

Most participants had a positive experience with the in-ear system, describing it as comfortable and user-friendly (90%, 26/29). In particular, during the nap tests, about 70% of the subjects (20/29) managed to fall asleep wearing both scalp and in-ear EEG systems. In a small number of cases (10%, 3/29), participants reported some physical discomfort with earplugs, primarily due to added pressure around the tragus and antitragus, especially noticeable during naps, which sometimes hindered their ability to fall asleep. One participant (subject 22) experienced the earpieces slipping out when turning onto their sides during sleep, leading him to remove and reinsert them. Regarding data acquisition, dual in-ear and scalp EEG recordings were successfully performed without technical issues in 29 out of 30 subjects. However, in one participant (subject 7), we encountered a problem with the in-ear acquisition system, which prevented the whole data acquisition. Additionally, in 7 subjects, interruptions occasionally occurred in the in-ear signal recordings during the nap protocol due to Bluetooth communication issues, requiring a system reboot.

### 4.2 Electrode skin contact impedance

Impedance spectra of the in-ear dry electrodes were recorded immediately after insertion (t0), after 10 min (t0 + 10 min), after 1 h (t0 + 1 h), and after 3 h (t0 + 3 h). At t0, we found that the impedance was relatively high (mean 902 ± 400 kΩ), with large inter-subject variations, but strongly decreases over time across the frequency range from 0.1 to 1,000 Hz, particularly after 1 h, and stabilizes around 3 h (Figure 2B). This trend indicates that the electrode-skin interface improves over time, possibly due to sweating and better electrode contact. Additionally, impedance was significantly decreased from t0 to t0 + 10 min, with further reductions at t0 + 1 h and t0 + 3 h (Figure 2C). The average impedance at 50 Hz across all intervals was 290.4 ± 95 kΩ, comparable to the impedance of a state-of-the-art in-ear dry electrode (for example, 377 kΩ in Kaveh et al., 2020). This data confirms that the used in-ear electrodes stabilize over time, achieving impedance values similar to those observed with other dry electrodes, thereby enhancing signal quality.

### 4.3 Visual inspection of in-ear signals

**Figure 3:**
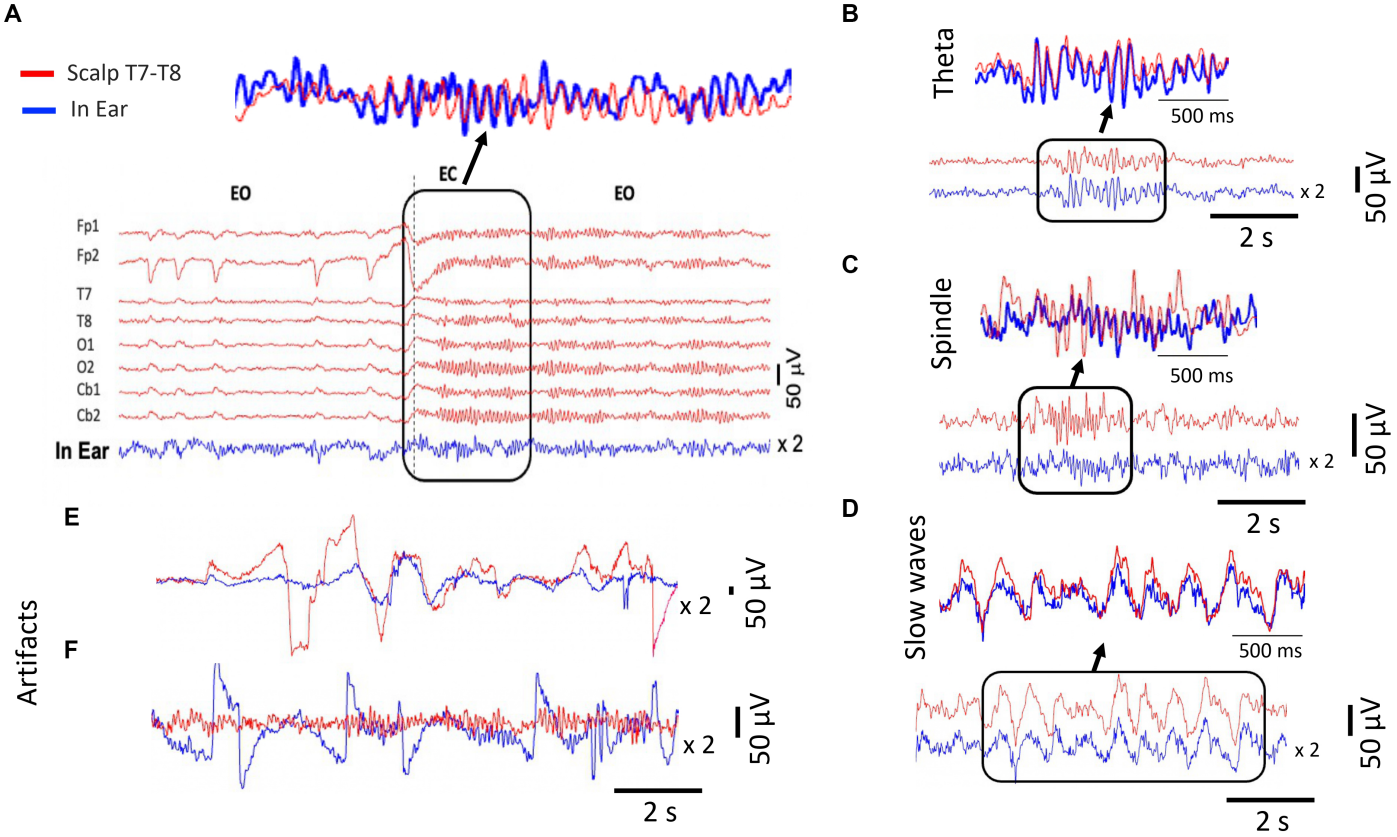
Signal analysis of different EEG waveforms from simultaneous scalp (T7-T8) and dry-contact in-ear recordings. The in-ear EEG signals are amplified (x2) for comparison. **(A)** Example of EEG signal traces comparing simultaneous scalp and in-ear recordings during “alpha test. The signals show epochs of eye-open (EO) and eye-closed (EC) states. The top panel highlights a detailed waveform comparison during the EC state, demonstrating the high similarity in alpha waves during the EC condition captured by the two systems (dry-contact in-ear in blue and gel-based scalp electrode cb2). **(B)** Comparison of theta wave activity (4–7 Hz) between scalp (red) and in-ear (blue) EEG recordings. **(C)** Comparison of slow wave activity (0.5–2 Hz) from the scalp (red) and in-ear (blue), highlighting the consistency in detected slow waves. **(D)** Spindle activity (12–16 Hz) from the scalp (red) and in-ear (blue), indicating the capability of the in-ear device to capture sleep spindles. **(E)** Example of movement artifacts (face and head) observed in in-ear (blue) EEG recordings compared to scalp (red). **(F)** Example of artifacts from poor skin contact observed in in-ear sensors (blue) during eyes closed (EC).

We initially evaluated the EEG signal quality through a systematic visual inspection of the filtered signals (0.3–35 Hz). In-ear and scalp EEG data were aligned and displayed on consecutive pages of 10-s portions. We utilized several standard electrode montages (referential on mastoids, longitudinal, and transversal bipolar montages) to inspect the scalp EEG. In most subjects (69%, 20 out of 29 cases), resting alpha waves with closed eyes at 8–12 Hz (posterior dominant rhythms) were confirmed on in-ear signals. Similar to standard scalp EEG, we observed in-ear alpha waves responsive to eye-opening and closure (Figure 3A). Furthermore, we evidenced high similarity between in-ear and scalp individual waveforms. Nevertheless, in-ear electrodes recorded smaller alpha waveforms than the scalp signal, with an average amplitude approximately two times lower. During the naps, theta waves (4–8 Hz) were identified in around 45% of cases of sleep (10/22), particularly during the transition from wakefulness to sleep, and were concurrent with T7-T8 waves in the same frequency range (Figure 3B). Additionally, during stages 2 and 3, spindles and slow waves were visually identified with shapes similar to scalp electrodes (Figures 3C,D). Once again, in-ear electrodes recorded smaller waveforms compared to the scalp signals.

This difference was particularly noticeable for spindles, which were often difficult to distinguish from background activity. Moreover, during nap recordings, involuntary head movements, mouth opening, or contractions of facial/jaw muscles frequently caused intermittent high voltage artifacts in the EEG signals. Signal inspection revealed isolated fluctuations in in-ear and scalp signals, with strong electrical potentials approximately 5–10 times the amplitude of brain signals (Figure 3E). Occasionally, we observed artifacts only in the in-ear signal. We identified contributors to these activities associated with ear canal deformations resulting from mandible movements (e.g., swallowing, mouth-opening, speaking) or changes in ear pressure when the subject laid their ears on the pillow. In such cases, poor skin contact with the in-ear sensors could also lead to significant fluctuations in EEG signals exceeding 100 μV (Figure 3F).

### 4.4 Evaluations in the time domain

#### 4.4.1 Root mean square values of the in-ear and scalp EEG signals

**Figure 4:**
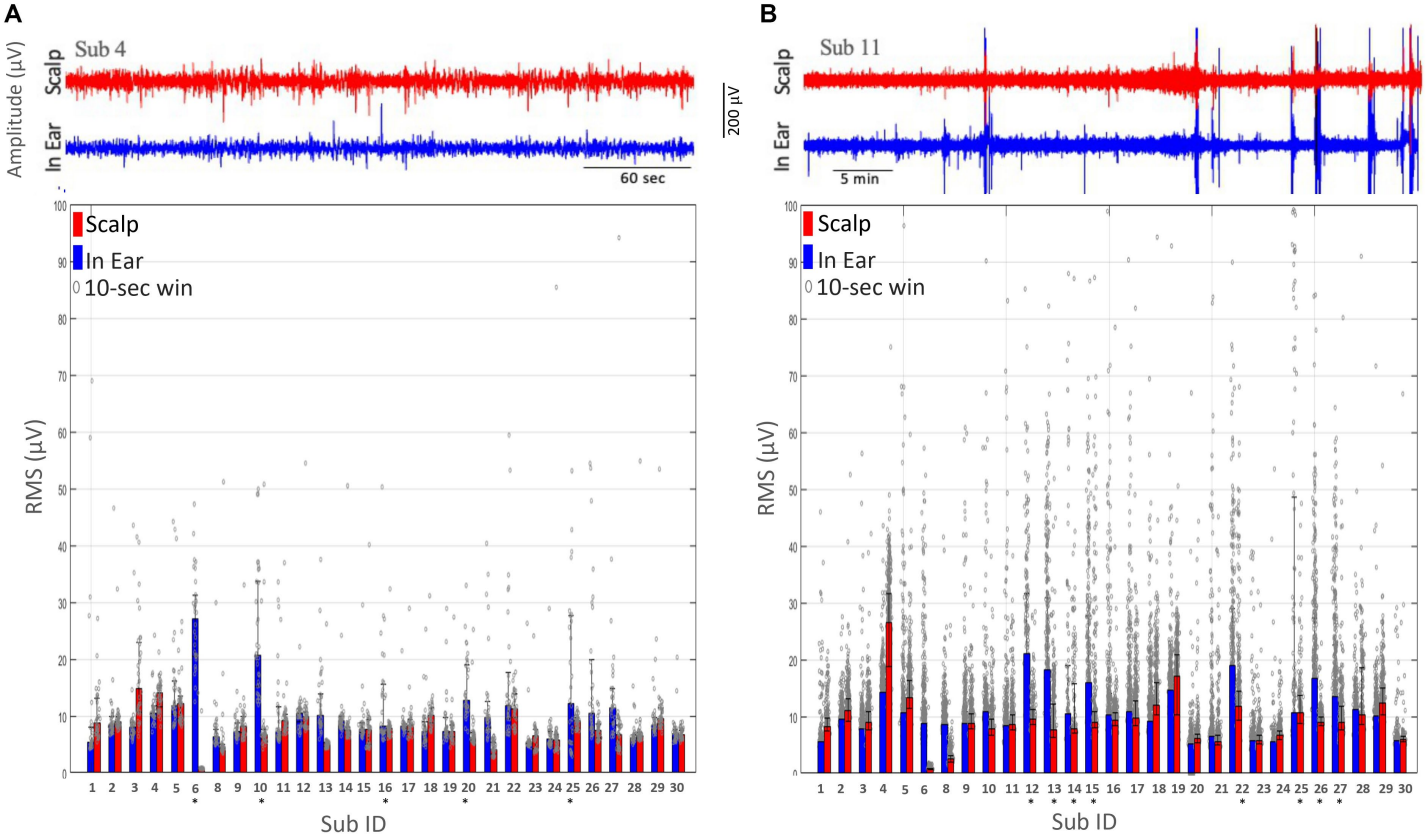
Comparison of root mean square (RMS) measurements obtained from scalp (T7-T8) and dry-contact in-ear electrodes. Individual RMS values are indicated by grey dots, with the mean RMS values represented by the height of the bars. Subjects marked with an asterisk (*) indicate those with notable differences between scalp and in-ear RMS values. **(A)** The RMS of the EEG signal amplitude during the alpha test is plotted for each subject (Sub ID) using scalp (red bars) and in-ear (blue bars) electrodes over 10-s windows. **(B)** The RMS of the EEG signal amplitude during the nap test plotted for each subject (Sub ID) using scalp (red bars) and in-ear (blue bars) electrodes over 10-s intervals.

During the “alpha tests,” we observed that the RMS values of the in-ear device remained consistently stable across the population and exhibited minor fluctuations from 5.3 μV to 28.8 μV (mean 12.3 ± 6.4 μV, see Figure 4A for individual RMS values), which fell within a comparable range to the simultaneously recorded scalp T7-T8 EEG signals using electrodes with gel (mean: 8.6 ± 3.5 μV; range: 0.76 μV - 20.2 μV). Comparing the distributions of intraindividual RMS values for ear-EEG and scalp-EEG, we found that a small number of the in-ear recordings, around 20% (5 out of 29, indicated by asterisks on Figure 4A) exhibited minor statistical differences between medians (right-tail Wilcoxon rank sum test, *p* > 0.05).

During the “nap tests,” we observed that the RMS values of the in-ear device displayed stronger fluctuations across the population, ranging from 5.2 μV to 21.0 μV (mean 10.8 ± 4.3 μV, as shown in Figure 4B), in comparison to the “alpha tests. Additionally, the simultaneously recorded scalp T7-T8 EEG signals using electrodes with gel exhibited comparable large fluctuations (range: 0.8 μV to 26.5 μV; mean: 9.4 ± 4.6 μV). However, in 27% (8 out of 29) of the subjects (as indicated by asterisks in Figure 4B), stronger discrepancies in the RMS values were observed between ear-EEG and scalp-EEG, suggesting a higher degree of in-ear signal degradation, possibly associated with intermittent lack of contact between the earplugs and the skin. In one particular instance (see subject 22 in Figure 4B), it was confirmed that the earpiece had fallen out of the ear during sleep and was repositioned by the subject in an unstable position.

#### 4.4.2 Proportion of artifacts

**Figure 5:**
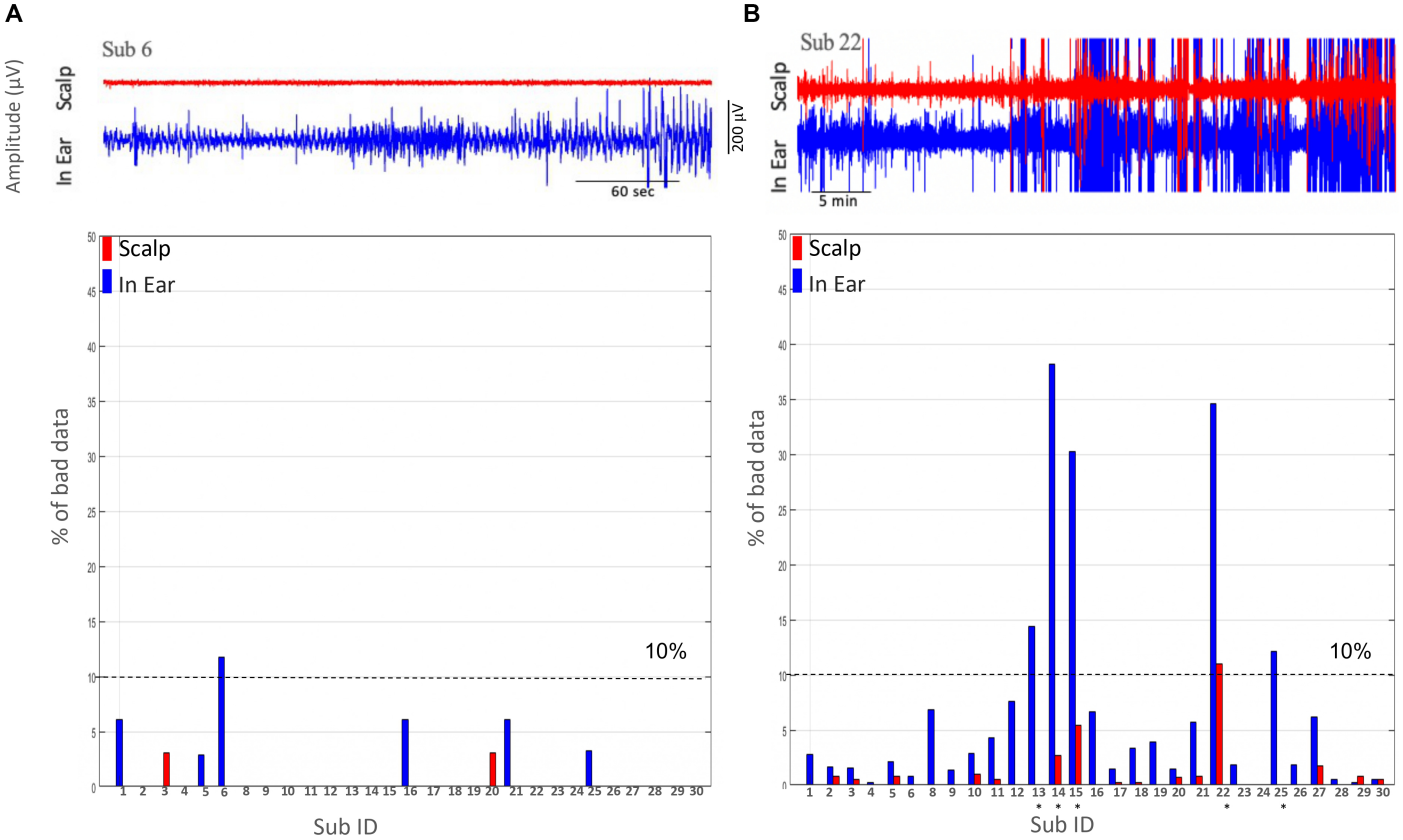
Comparison of the percentage % of bad data between scalp (T7-T8) and in-ear EEG recordings. **(A)** The percentage of bad data segments for each subject using scalp (red) and in-ear (blue) EEG recordings during the alpha test. The dashed line at 10% indicates the threshold for acceptable data quality. Subjects are identified by their ID numbers along the x-axis. Most subjects show low percentages of bad data, with a few exceptions. **(B)** The percentage of bad data segments for a different group of subjects, again comparing scalp (red) and in-ear (blue) EEG recordings. The 10% threshold line is included for reference. Notable differences (subjects marked by asterisks) in bad data percentages are observed, especially for certain subjects (e.g., Sub 13 and Sub 22).

Existing literature on EEG artifacts suggested that segments with extreme values (±200 μV) can be regarded as low-quality data (Islam et al., 2016). To identify transient artifacts in EEG segments, we quantified epochs of 10 s (non-overlapping) that exceeded the threshold of −100 μV to +100 μV. In a consistent way with the results for the root mean square values during the “alpha tests,” we observed that in-ear signals have a very small proportion of bad data, with only a single subject exhibiting bad data exceeding 10% of the entire recording, which is comparable to the simultaneously recorded scalp T7-T8 EEG signals (Figure 5A). However, during the “nap tests,” in 17% (5 out of 29) of subjects (as indicated by asterisks in Figure 5B), we identified a significant proportion of bad data (>10%) for the ear-EEG, suggesting intermittent degradation of the in-ear signals.

### 4.5 Evaluations in frequency domain

#### 4.5.1 Alpha peak criteria for the eye closed condition

**Figure 6:**
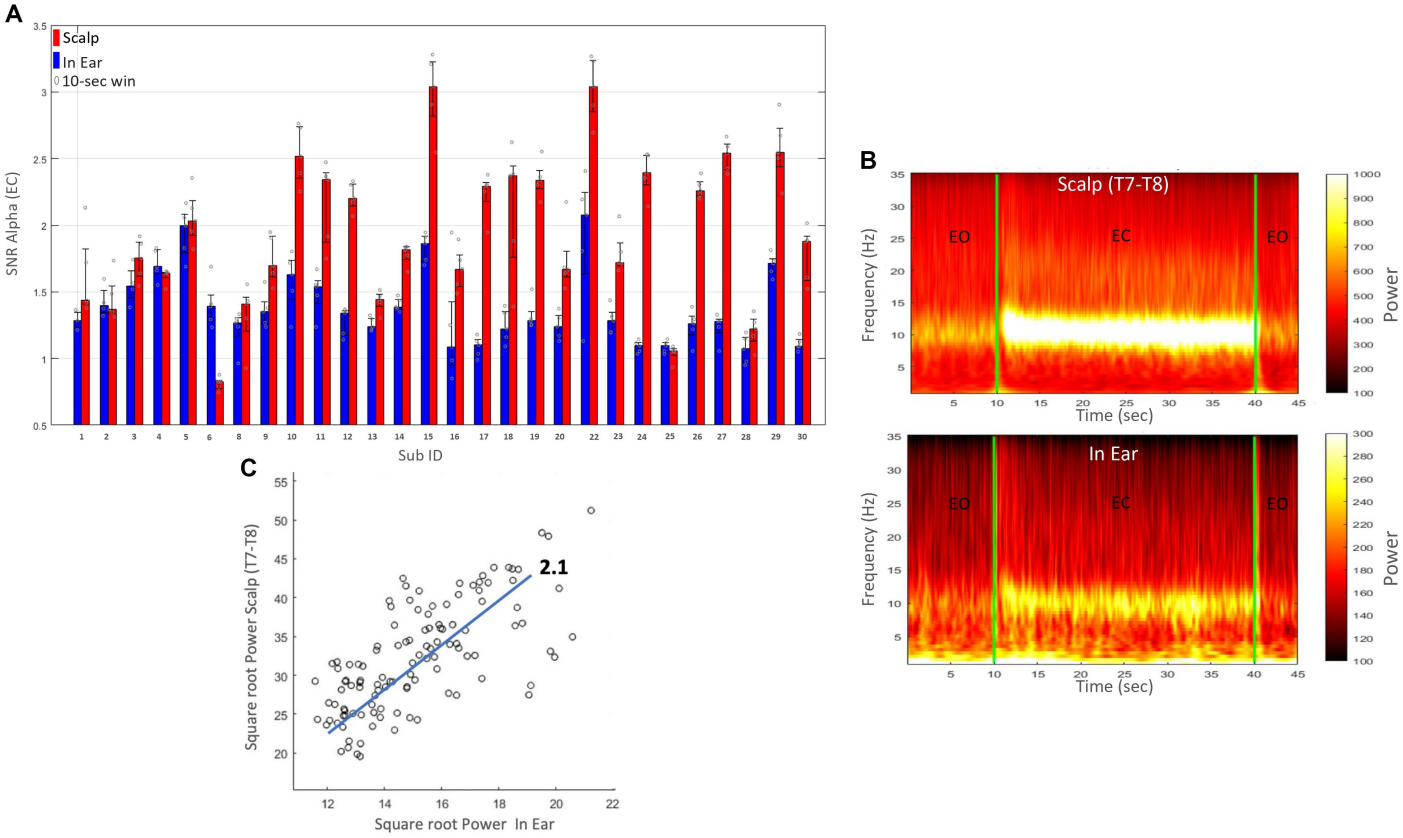
**(A)** Signal-to-noise ratio (SNR) for the alpha band (8–12 Hz) during the eyes-closed (EC) condition for each subject (Sub ID). Red bars represent scalp (T7-T8) recordings, and blue bars represent dry-contact in-ear recordings. Error bars indicate the standard error. **(B)** Spectrograms of EEG recordings from in-ear electrodes (bottom) and simultaneously recorded scalp T7-T8 electrodes (top), averaged across all subjects. Periods of eyes open (EO) and eyes closed (EC) are indicated. **(C)** Scatter plot showing the correlation between the square root of the power recorded from scalp (T7-T8) and in-ear electrodes. The x-axis represents the square root of the power from in-ear electrodes, and the y-axis represents the square root of the power from scalp (T7-T8) electrodes. Each point corresponds to a 10-s epoch. A linear trend line with a slope of 2.1 is included.

During the “alpha test,” subjects were instructed to close their eyes every 30 s over 5-min periods. In the condition where participants had their eyes closed, we utilized the data to determine the individual frequency peak within the traditional alpha frequency range (8–12 Hz). We employed a decomposition of Gabor wavelets to calculate the time-frequency spectrograms during the eye-closed condition. Figure 6B illustrates the time-frequency spectrograms of the in-ear EEG and the simultaneously recorded scalp T7-T8 (grand average over all subjects and eye closings). Notice the prominent peaks in the alpha range during eye closings in both in-ear and scalp signals. Furthermore, we compared the power amplitudes of alpha waves recorded on both devices, evaluating their similarity with the linear regression coefficient. Our findings revealed that scalp electrodes recorded higher alpha power, averaging around twice that of in-ear electrodes (Figure 6C).

On average, we found that the SNR of alpha waves had values of 1.4 ± 0.3 (range: 1–2.2) and exceeded 1.5 in 29% (8/28) of cases (Figure 6A). These values were comparable to those reported in research laboratories, indicating that for dry in-ear sensors, the ratio for alpha power typically exceeds 1.5 (Mandekar et al., 2022). In 21% (6/28), SNR(alpha) values were close to 1, suggesting negligible responses in the alpha frequency range. Furthermore, in 5 out of 6 of these cases, high SNR(alpha) values were observed at the scalp (>1.5), suggesting a degradation of the in-ear signals in these instances. As expected, the scalp T7-T8 signals demonstrated a slightly higher SNR than the in-ear EEG signal (with an average of 1.9 ± 0.6, range: 1–3.0).

#### 4.5.2 Alpha wave correlations during the eye closed condition

**Figure 7:**
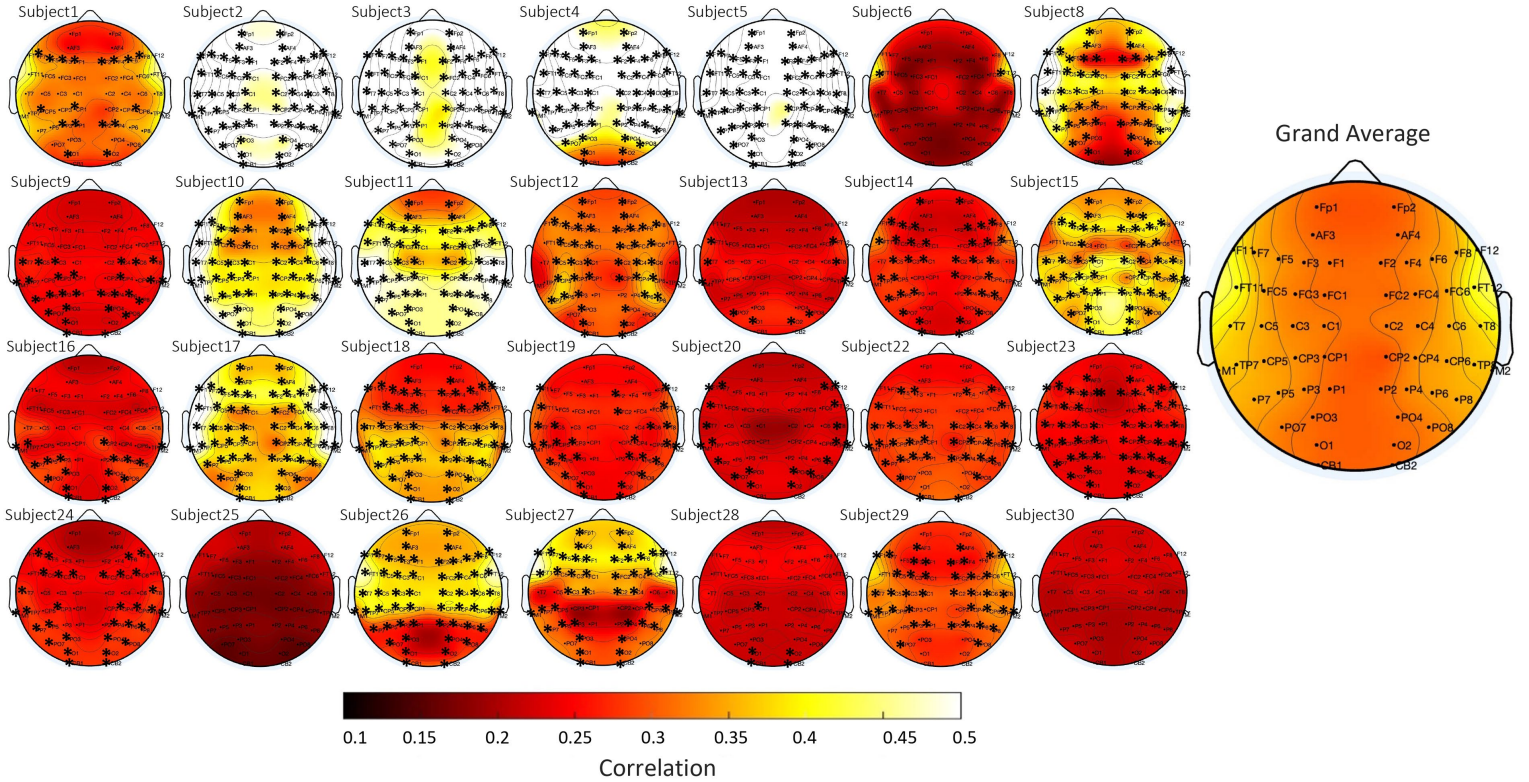
Topographical plots of alpha wave correlation values during the eyes-closed condition, comparing scalp (contralateral bipolar montage) and in-ear EEG signals for each subject. Each subplot corresponds to a different subject, with scalp electrode locations indicated. The color scale represents correlation values, ranging from 0.1 (dark red) to 0.5 (yellow). The grand average across all subjects is displayed on the right.

Statistical analysis revealed that 93% of subjects (26 out of 28) exhibited a significant (*p* < 0.01, Pitman nonparametric permutation test) correlation between scalp and in-ear signals (see Figure 7 for individual results and grand average). We found the strongest correlations between the in-ear signal and the scalp EEG at FT11-FT12 (0.42 ± 0.02 on average) and T7-T8 (0.38 ± 0.01 on average), compared to other parietal electrodes (P1-P2; 0.3 ± 0.01 on average) (refer to Figure 7 for global average). Only 2 subjects had a negligible or small correlation (<0.2). The high correlation observed between the in-ear and scalp electrodes positioned at temporal locations implies that most sources recorded with electrodes in contralateral ear canals originate from the temporal lobe.

#### 4.5.3 Power spectra

**Figure 8:**
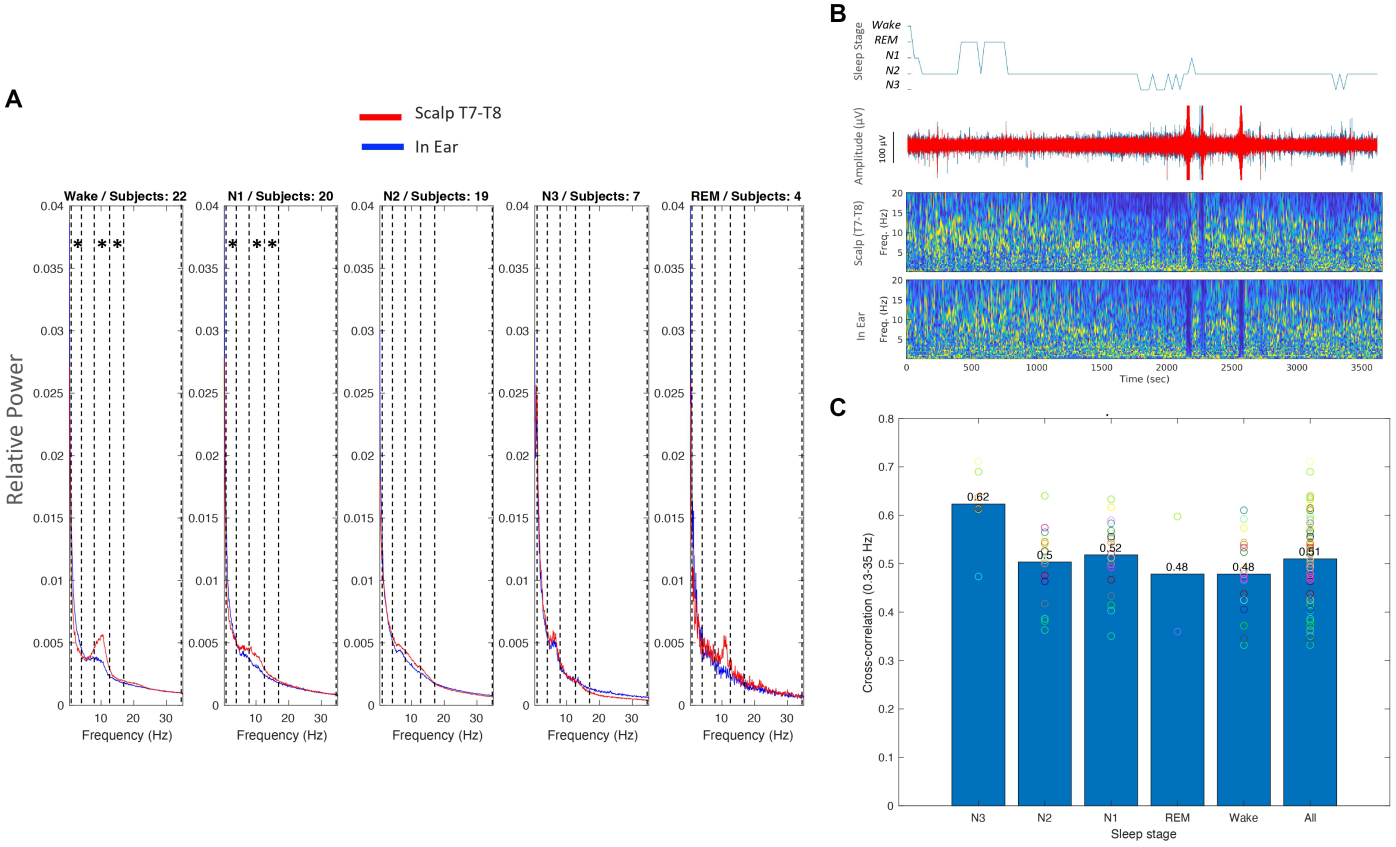
**(A)** Relative spectral power (RSP) across subjects for different sleep stages (Wake, N1, N2, N3, REM) for scalp (red) and in-ear (blue) EEG recordings. Each plot represents the average RSP across subjects for the specified sleep stage. Asterisks inside the vertical dashed lines indicate the frequency bands where there are statistically significant differences (*p* < 0.01). **(B)** An example hypnogram of a nap (top), raw signals (middle), and relative spectrograms of scalp (T7-T8) and in-ear EEG signals (bottom) during sleep. **(C)** Cross-correlation values between scalp (T7-T8) and in-ear EEG signals across different sleep stages. Bars represent mean cross-correlation values, with individual subject values overlaid as circles. The overall mean across all sleep stages is shown on the right.

We quantified the evolution of relative spectral power (RSP) of scalp (T7-T8) and in-ear EEG signals across different sleep stages (Wake, N1, N2, N3, and REM). We calculated corresponding RSP values within standard frequency ranges (Delta: 0.3–4 Hz, Theta: 4–8 Hz, Alpha: 8–12 Hz, Beta1: 12–18 Hz, and Beta2: 18–35 Hz). Once again, we considered in our analysis the underwent artifact rejection criteria, where only windows that did not exceed the threshold of −100 μV to +100 μV (for at least 10% of the time). Across sleep stages, the spectral characteristics of in-ear recordings revealed clear similarities with scalp EEG T7-T8 channels (Figure 8A for the grand average). During Wake, the RSP confirmed a single peak around 10 Hz, approximately aligned across in-ear and scalp channels.

Nevertheless, the relative alpha power was significantly higher on the scalp compared to the in-ear recordings (p < 0.01, Pitman nonparametric permutation test). Further statistical differences between scalp and in-ear channels were also identified during Wake in the Delta and Beta1 frequency ranges. In contrast, during N2 and N3, power levels remained strongly similar between in-ear and scalp recordings (with no statistically significant differences in N2). Here, low-frequency activities (Delta and Theta) prevailed over all other frequency bands (Figure 8B for an illustrative subject). In particular, during N3, a strong peak between 4 and 7 Hz (theta range) was identified in around 45% of sleep cases (10 out of 22) with similar power in both scalp and in-ear channels.

#### 4.5.4 Correlations between in-ear and scalp EEG during sleep stages

To further explore similarities between individual in-ear and scalp patterns, we estimated correlation coefficients between the in-ear signal and the scalp EEG (T7-T8) during sleep stages. Across the 22 subjects, we found a significant correlation between scalp and in-ear signals in all cases (100%), with an average correlation of 0.51 ± 0.086. This correlation increased across different sleep stages (Figure 8C). During Wake, N1, and N2, the correlation coefficients had high values (mean 0.48 ± 0.08, 0.52 ± 0.07, and 0.5 ± 0.07, respectively), consistently indicating moderate coherence between in-ear and scalp signals. Interestingly, the N3 stage did exhibit a notable increase in coherence, with a substantial correlation between in-ear and scalp channels, averaging a mean value of 0.62 ± 0.08. This suggests a potential synchronization of slow-wave activity between scalp and in-ear EEG signals during deep sleep. Finally, during REM sleep, the correlation trends showed moderate coherence between both signals across all frequency bands (0.48 ± 0.17 on average), suggesting slight differences in EEG patterns between the two recording modalities during this stage.

## 5 Discussion

In our study, we conducted a comprehensive signal quality analysis of a mobile in-ear EEG device with dry-contact, comparing it to a standard research-grade EEG system employing wet electrodes. Our investigation first focused on impedance changes of the skin-electrode interface in the ear canal, revealing a progressive decrease over time, particularly notable after 1 h of use. This decline indicates an improved electrode-skin interface and enhanced signal quality, consistent with previous research in dry sensors (Kaveh et al., 2020; Xu et al., 2023). Next, to quantitatively evaluate signal quality, we employed several measures, including RMS values, % of good data, SNR (alpha), and cross-signal correlations in the alpha band during eye-closed conditions or in the broadband during sleep. Comparisons with scalp T7-T8 signals indicated high performance, particularly during quiet resting states when participants minimized head and facial muscle contractions. In particular, alpha waves were detected in approximately 80% of in-ear signals, exhibiting a strong correlation with scalp electrodes albeit with slightly lower amplitude. Also, during sleep, we found a relatively high correlation (between 0.48 and 0.62) regardless of the sleep phases, indicating that the signals were generally consistent between in-ear and scalp during the whole sleep session. Throughout our analysis, we found clear similarities in the temporal and spectral characteristics between scalp and in-ear signals. These results are consistent with other studies in the literature comparing the similarity between scalp T7 and T8 and in-ear signals (Looney et al., 2011). In line with additional research using full scalp coverage (Mikkelsen et al., 2015; Mikkelsen et al., 2017b), we found the highest correlation values in temporal regions. This suggests that the primary sources of the signals captured by electrodes in the ear canals are located within both temporal lobes, a conclusion also supported by neural source modeling studies (Meiser et al., 2020; Yarici et al., 2023). However, it is important to note that most previous studies have validated the similarity of in-ear and scalp EEG signals based on averaged responses, such as event-related potentials (ERP) or steady-state responses (Kappel et al., 2019). These studies typically do not account for individual brain waves, which are a standard focus in traditional EEG analysis. For instance, research like the study by Mikkelsen et al. (2015) showed that in-ear EEG performs comparably to conventional scalp channels located close to the ears in spectrogram-based analysis and in detecting similar timings of ERP components. Similarly, in sleep research, previous studies that investigated the specific use of in-ear electrodes have primarily focused on macrostructure features that characterize sleep, such as the comparison of sleep staging or sleep timing parameters (Mikkelsen et al., 2017a; Tabar et al., 2023). Following a different approach, our study is one of the first to investigate the point-by-point correlation of waveform similarity between in-ear and scalp EEG systems during wakefulness and sleep. By demonstrating that EEG systems can effectively correlate with each other in spontaneous EEG waves, our research sets a standard for future assessments in clinical settings, allowing clinicians to make more informed decisions based on consistent and reliable interpretations of in-ear EEG data. Additionally, while our study focused on linear correlation, future research could benefit from incorporating non-linear similarity metrics, such as mutual information or conditional entropy (Hlaváčková-Schindler et al., 2007). These metrics can help address possible non-linear warping and distortions of the in-ear signals, thus providing a more comprehensive depth to the comparisons.

Our study further affirmed that dry electrodes, known for their ease of use and reusability, are well-suited for wearable in-ear EEG devices designed for long-term monitoring. However, the EEG signals recorded by these dry electrodes are more susceptible to interference from movement-related artifacts due to their typically high skin-electrode impedance. The electrodes showed high sensibility to electrical artifacts, such as electrode pops emerging from abrupt impedance changes, which are visually recognizable in the EEG as very large, abrupt, sharp artifacts. In contrast, conventional wet electrodes mitigate this problem by being glued to the scalp. The conductive paste also acts as a shock absorber, minimizing relative motion between the electrode and scalp, thereby reducing motion artifacts. Here, unlike other commercially available systems (e.g., MN8 earbuds from Emotiv®), the used in-ear device incorporates active electrodes with a high input impedance, effectively shielding the signal from external electromagnetic interference and thereby enhancing signal quality. Nevertheless, as reported, especially during Wake or N1 stages, where spontaneous head and facial movements were present, the performance of in-ear recordings was marginally compromised (bad data >10% of the recording) in approximately 30% of subjects due to movements causing deformation of the ear canal and interruptions in skin contact. These signal degradations were considerably influenced by the stability of the contact surface inside the ear canal. Variable contact surfaces may occur in different individuals depending on multiple parameters, including the geometrical complexity of the ear canal and related contact pressure. Therefore, a significant factor contributing to signal quality could be the fit of the electrodes within the ear canal, which differs among subjects. Additionally, inherent physiological and anatomical differences further compound these variations. These differences can influence the effectiveness of the electrodes in capturing electrical activity, thereby affecting the overall correlation between in-ear and scalp EEG results.

Thus, addressing this variability is challenging due to the small sample size, which included only 29 subjects. While the results are indicative, they are not definitive, and further validation through larger-scale studies is necessary in the future. Additionally, the method of simultaneous in-ear and scalp recordings presents challenges due to the spatial scale at which EEG signals change over the scalp’s surface. Cortical activity dynamics involve propagating waves at various frequencies that alter the signal’s amplitude at a sub-centimeter scale in the brain (Ryynanen et al., 2006). Consequently, it is unrealistic to expect that electrodes inside the ear canal would record the exact same signal as those on the scalp. This is likely the cause for the high variability of the cross-correlations reported in this article. Additionally, our results show that the alpha power recorded by scalp electrodes was, on average, approximately twice as high as that captured by in-ear electrodes (see Figures 6C, 8A). This consistently higher amplitude of alpha waves at the scalp is likely due to the closer proximity of scalp electrodes to the brain’s electrical sources and reduced interference from barriers, such as skull bone and tissue, compared to in-ear electrodes. These differences highlight the unique characteristics and potential limitations of each electrode type and suggest that in-ear EEG technology may require specific calibration or enhancements to match the performance of traditional scalp-based systems. Such insights are essential for developing more accurate and reliable EEG monitoring techniques adaptable to various clinical and research settings. Furthermore, this highlights the importance of tailored approaches in the application of ear-EEG technology, emphasizing the need for individualized considerations. Recognizing and addressing this variability is crucial to ensure the accuracy and reliability of in-ear EEG measurements across diverse individuals and populations. Further exploration and refinement of the technology are necessary to address these challenges and optimize its effectiveness in various research and clinical contexts. This approach could include creating customized algorithms or de-noising techniques specifically tailored to handle individual users’ artifacts, such as tongue movements, eye movements, and chewing. Additionally, innovations in electrode design and strategic placement, derived from the fields of hearing aid technology or ear, nose, and throat (ENT) medicine, could further enhance the effectiveness of these solutions. In this context, recent research focuses on the materials used for electrodes and the design of earpieces, emphasizing the shape of the electrodes or sensors with highly flexible or adaptable components (Wang et al., 2023). Further design improvements, such as earpieces that stay in place or sensors that adapt to changes in the ear’s shape, will enhance in-ear recording for long-term, out-of-lab use. In that sense, obtaining precise anatomical data of participants’ ear canals before device deployment allows for the tailoring of electrode design and placement, ensuring optimal fit and contact with the skin, thus minimizing signal artifacts and maximizing signal quality. However, previous work has shown that the signal quality from customized earpieces does not significantly surpass that of generic earpieces (Kidmose et al., 2013). Device developers might also consider incorporating feedback mechanisms into in-ear EEG devices to monitor electrode-skin contact and signal quality during data acquisition continuously. This could include integrating sensors or impedance monitoring systems into the device design to detect and address issues related to poor electrode contact or signal degradation in real time. In addition to technological improvements, standardized electrode placement and signal acquisition protocols could ensure consistency and reproducibility across participants. Providing training and guidelines for device fitting and positioning to research participants or healthcare professionals responsible for device deployment could further enhance the reliability of in-ear EEG measurements.

Clearly, signal distortions or degradations observed in in-ear EEG measurements could impact the accuracy of assessing brain activity, particularly in clinical settings where precise measurements are imperative. Discrepancies in signal amplitudes between scalp and in-ear EEG recordings may pose challenges in establishing standardized protocols and reference ranges for interpreting EEG data. Clinicians and researchers may need to account for these discrepancies when utilizing in-ear EEG devices for longitudinal monitoring or cross-sectional studies. Further research and validation studies are needed to understand the implications of these disparities comprehensively and to optimize the application of in-ear EEG technology in clinical practice.

## 6 Conclusion

Our results not only prove the applicability of a novel in-ear device for EEG acquisition but also hint at the exciting potential of this technology. We found no considerable differences in signal characteristics compared to gel-based T7-T8 scalp EEG electrodes, despite marginal effects due to deformations of the ear canal. The overall data quality of EEG earbuds was positive, and the in-ear system offers increased comfort, particularly for repetitive dry-contact biopotential measurement applications and durations longer than 60 min. These findings suggest that sleep monitoring with EEG earbuds will be feasible in the majority of cases, opening up a world of possibilities for this technology.

Future research should focus on improving in-ear device design to minimize signal disruptions during movement and optimize sensor placement. To expand its utility, further exploration of clinical applications, especially in sleep monitoring and real-world settings, is warranted.

## Data Availability

The raw data supporting the conclusions of this article will be made available by the authors, without undue reservation.

## References

[ref1] Athavipach C. Pan-Ngum S. Israsena P. (2019). A wearable in-ear EEG device for emotion monitoring. Sensors (Basel, Switzerland) 19:E4014. doi: 10.3390/s19184014 PMC6767669 31533329

[ref2] Benjamini Y. Hochberg Y. (1995). Controlling the false discovery rate: a practical and powerful approach to multiple testing. J. R. Stat. Soc. B (Methodological) 57, 289–300. doi: 10.1111/j.2517-6161.1995.tb02031.x

[ref3] Berry R. Quan S. Abreu A. (2020). The AASM manual for the scoring of sleep and associated events: Rules, terminology and technical specifications, version 2.6. Darien: American Academy of Sleep Medicine.

[ref4] Butar B. F. Park J. W. (2008). Permutation tests for comparing two populations. J. Math. Sci. Math. Educ. 3, 19–30.

[ref5] Chi Y. M. Jung T.-P. Cauwenberghs G. (2010). Dry-contact and noncontact biopotential electrodes: methodological review. IEEE Rev. Biomed. Eng. 3, 106–119. doi: 10.1109/RBME.2010.2084078 22275204

[ref6] Christensen C. B. Harte J. M. Lunner T. Kidmose P. (2018). Ear-EEG-based objective hearing threshold estimation evaluated on Normal hearing subjects. IEEE Trans. Biomed. Eng. 65, 1026–1034. doi: 10.1109/TBME.2017.2737700 28796603

[ref7] Correia G. Crosse M. J. Valdes A. L. (2024). Brain wearables: validation toolkit for ear-level EEG sensors. Sensors 24:1226. doi: 10.3390/s24041226 38400384 PMC10893377

[ref8] Daniel W. W. Cross C. L. (2018). Biostatistics a Foundation for Analysis in the health sciences. 10th Edn. Hoboken, NJ: Wiley.

[ref9] Erickson B. Rich R. Shankar S. Kim B. Driscoll N. Mentzelopoulos G.. (2024). Evaluating and benchmarking the EEG signal quality of high-density, dry MXene-based electrode arrays against gelled ag/AgCl electrodes. J. Neural Eng. 21:016005. doi: 10.1088/1741-2552/ad141e, PMID: 38081060 PMC10788783

[ref10] Goverdovsky V. Looney D. Kidmose P. Mandic D. P. (2016). In-ear EEG from viscoelastic generic earpieces: robust and unobtrusive 24/7 monitoring. IEEE Sensors J. 16, 271–277. doi: 10.1109/JSEN.2015.2471183

[ref11] Goverdovsky V. von Rosenberg W. Nakamura T. Looney D. Sharp D. J. Papavassiliou C.. (2017). Hearables: multimodal physiological in-ear sensing. Sci. Rep. 7:6948. doi: 10.1038/s41598-017-06925-2 28761162 PMC5537365

[ref12] Haaga K. A. Datseris G. (2022). TimeseriesSurrogates.Jl: a Julia package for Generatingsurrogate data. J. Open Source Softw. 7:4414. doi: 10.21105/joss.04414

[ref13] Hlaváčková-Schindler K. Palus M. Vejmelka M. Bhattacharya J. (2007). Causality detection based on information-theoretic approaches in time series analysis. Phys. Rep. 441, 1–46. doi: 10.1016/j.physrep.2006.12.004

[ref14] Hong S. Kwon H. Choi S. H. Park K. S. (2018). Intelligent system for drowsiness recognition based on ear canal electroencephalography with Photoplethysmography and electrocardiography. Inf. Sci. 453, 302–322. doi: 10.1016/j.ins.2018.04.003

[ref15] Islam M. K. Rastegarnia A. Yang Z. (2016). Methods for artifact detection and removal from scalp EEG: a review. Clin. Neurophysiol. 46, 287–305. doi: 10.1016/j.neucli.2016.07.002 27751622

[ref16] Joyner M. Hsu S. H. Martin S. Dwyer J. Chen D. F. Sameni R.. (2024). Using a standalone ear-EEG device for focal-onset seizure detection. Bioelectron Med. 10:4. doi: 10.1186/s42234-023-00135-0, PMID: 38321561 PMC10848360

[ref17] Juez J. Y. Moumane H. Nassar M. Molina-Salcedo I. Segura-Quijano F. E. Valderrama M.. (2024). Ear-EEG devices for the assessment of brain activity: a review. IEEE Sens. J. 1:668. doi: 10.1109/JSEN.2024.3415668

[ref18] Kaongoen N. Choi J. Choi J. W. Kwon H. Hwang C. Hwang G.. (2023). The future of wearable EEG: a review of ear-EEG technology and its applications. J. Neural Eng. 20:051002. doi: 10.1088/1741-2552/acfcda 37748474

[ref19] Kappel S. L. Kidmose P. (2015). Study of impedance spectra for dry and wet EarEEG electrodes. Annu. Int. Conf. IEEE Eng. Med. Biol. Soc. 2015, 3161–3164. doi: 10.1109/EMBC.2015.7319063, PMID: 26736963

[ref20] Kappel S. L. Rank M. L. Toft H. O. Andersen M. Kidmose P. (2019). Dry-contact electrode ear-EEG. IEEE Trans. Biomed. Eng. 66, 150–158. doi: 10.1109/TBME.2018.2835778 29993415

[ref21] Kaveh R. Doong J. Zhou A. Schwendeman C. Gopalan K. Burghardt F.. (2020). *Wireless user-generic ear EEG*. arXiv. Available at: http://arxiv.org/abs/2003.00657. 10.1109/TBCAS.2020.3001265 32746342

[ref22] Keil A. Debener S. Gratton G. Junghöfer M. Kappenman E. S. Luck S. J.. (2014). Committee report: publication guidelines and recommendations for studies using electroencephalography and magnetoencephalography. Psychophysiology 51, 1–21. doi: 10.1111/psyp.12147 24147581

[ref23] Kidmose P. Looney D. Mandic D. P. (2012). Auditory evoked responses from ear-EEG recordings. Annu. Int. Conf. Eng. Med. Biol. Soc. 2012, 586–589. doi: 10.1109/EMBC.2012.6345999, PMID: 23365960

[ref24] Kidmose P. Looney D. Ungstrup M. Rank M. L. Mandic D. P. (2013). A study of evoked potentials from ear-EEG. IEEE Trans. Biomed. Eng. 60, 2824–2830. doi: 10.1109/TBME.2013.2264956 23722447

[ref25] Landis J. R. Koch G. G. (1977). The measurement of observer agreement for categorical data. Biometrics 33:159. doi: 10.2307/2529310 843571

[ref26] Lee J. H. Ma S. Remaley J. Gamper H. Holbery J. D. Tashev I.. (2020). *Stress monitoring using multimodal bio-sensing headset*. In: Extended abstracts of the 2020 CHI conference on human factors in computing systems, Honolulu HI USA: ACM, pp. 1–7.

[ref27] Looney D. Park C. Kidmose P. Rank M. L. Ungstrup M. Rosenkranz K.. (2011). An in-the-ear platform for recording electroencephalogram. Annu Int Conf IEEE Eng Med Biol Soc 2011, 6882–6885. doi: 10.1109/IEMBS.2011.6091733, PMID: 22255920

[ref28] Lopes F. Teixeira C. A. Vollmar C. Le Van Quyen M. Gotman J. (2023). Removing artefacts and periodically retraining improve performance of neural network-based seizure prediction models. Sci. Rep. 13:5918. doi: 10.1038/s41598-023-31940-9 37041158 PMC10090199

[ref29] Mandekar S. Holland A. Thielen M. Behbahani M. Melnykowycz M. (2022). Advancing towards ubiquitous EEG, correlation of in-ear EEG with forehead EEG. Sensors 22:1568. doi: 10.3390/s22041568, PMID: 35214468 PMC8879675

[ref30] Maris E. Oostenveld R. (2007). Nonparametric statistical testing of EEG- and MEG-data. J. Neurosci. Methods 164, 177–190. doi: 10.1016/j.jneumeth.2007.03.024 17517438

[ref31] Meiser A. Tadel F. Debener S. Bleichner M. G. (2020). The sensitivity of ear-EEG: evaluating the source-sensor relationship using forward modeling. Brain Topogr. 33, 665–676. doi: 10.1007/s10548-020-00793-2 32833181 PMC7593286

[ref32] Merrill N. Curran M. T. Gandhi S. Chuang J. (2019). One-step, three-factor Passthought authentication with custom-fit, in-ear EEG. Front. Neurosci. 13:354. doi: 10.3389/fnins.2019.00354, PMID: 31133772 PMC6524701

[ref33] Mikkelsen K. B. Kappel S. L. Mandic D. P. Kidmose P. (2015). EEG recorded from the ear: characterizing the ear-EEG method. Front. Neurosci. 9:438. doi: 10.3389/fnins.2015.00438 26635514 PMC4649040

[ref34] Mikkelsen K. B. Kidmose P. Hansen L. K. (2017b). On the keyhole hypothesis: high mutual information between ear and scalp EEG. Front. Hum. Neurosci. 11:341. doi: 10.3389/fnhum.2017.00341, PMID: 28713253 PMC5492868

[ref35] Mikkelsen K. B. Tabar Y. R. Kappel S. L. Christensen C. B. Toft H. O. Hemmsen M. C.. (2019). Accurate whole-night sleep monitoring with dry-contact ear-EEG. Sci. Rep. 9:16824. doi: 10.1038/s41598-019-53115-3 31727953 PMC6856384

[ref36] Mikkelsen K. B. Villadsen D. B. Otto M. Kidmose P. (2017a). Automatic sleep staging using ear-EEG. Biomed. Eng. Online 16:111. doi: 10.1186/s12938-017-0400-5 28927417 PMC5606130

[ref37] Morlet J. Arens G. Fourgeau E. Giard D. (1982). Wave propagation and sampling theory—part II: sampling theory and complex waves. Geophysics 47, 222–236. doi: 10.1190/1.1441329

[ref38] Musaeus C. S. Frederiksen K. S. Andersen B. B. Høgh P. Kidmose P. Fabricius M.. (2023a). Detection of subclinical Epileptiform discharges in Alzheimer’s disease using long-term outpatient EEG monitoring. Neurobiol. Dis. 183:106149. doi: 10.1016/j.nbd.2023.106149, PMID: 37196736

[ref39] Musaeus C. S. Kjær T. W. Hribljan M. C. Andersen B. B. Høgh P. Kidmose P.. (2023b). Subclinical Epileptiform activity in dementia with Lewy bodies. Mov. Disord. 2023:29531. doi: 10.1002/mds.29531 37431847

[ref40] Nichols T. E. Holmes A. P. (2002). Nonparametric permutation tests for functional neuroimaging: a primer with examples. Hum. Brain Mapp. 15, 1–25. doi: 10.1002/hbm.1058 11747097 PMC6871862

[ref41] Perslev M. Darkner S. Kempfner L. Nikolic M. Jennum P. J. Igel C. (2021). U-sleep: resilient high-frequency sleep staging. NPJ Digit. Med. 4:72. doi: 10.1038/s41746-021-00440-5 33859353 PMC8050216

[ref42] Röddiger T. Clarke C. Breitling P. Schneegans T. Zhao H. Gellersen H.. (2022). Sensing with Earables: a systematic literature review and taxonomy of phenomena. Proc. ACM Interact. Mob. Wearable Ubiquitous Technol. 6, 1–57. doi: 10.1145/3550314

[ref43] Ryynanen O. R. M. Hyttinen J. A. K. Malmivuo J. A. (2006). Effect of measurement noise and electrode density on the spatial resolution of cortical potential distribution with different resistivity values for the skull. IEEE Trans. Biomed. Eng. 53, 1851–1858. doi: 10.1109/TBME.2006.873744 16941841

[ref44] Shin J. H. Kwon J. Kim J. U. Ryu H. Jehyung Ok S. Kwon J.. (2022). Wearable EEG electronics for a brain–AI closed-loop system to enhance autonomous machine decision-making. NPJ Flexible Electron. 6:32. doi: 10.1038/s41528-022-00164-w

[ref45] Tabar Y. R. Mikkelsen K. B. Shenton N. Kappel S. L. Bertelsen A. R. Nikbakht R.. (2023). At-home sleep monitoring using generic ear-EEG. Front. Neurosci. 17:987578. doi: 10.3389/fnins.2023.987578, PMID: 36816118 PMC9928964

[ref46] Tautan A. M.. (2014). *Signal quality in dry electrode EEG and the relation to skin-electrode contact impedance magnitude*. In: Proceedings of the International Conference on Biomedical Electronics and Devices, pp. 12–22. ESEO, Angers, Loire Valley, France: SCITEPRESS - Science and and Technology Publications.

[ref47] Theiler J. Eubank S. Longtin A. Galdrikian B. Doyne Farmer J. (1992). Testing for nonlinearity in time series: the method of surrogate data. Phys. D Nonlinear Phenomena 58, 77–94. doi: 10.1016/0167-2789(92)90102-S

[ref48] Valentin O. Viallet G. Delnavaz A. Cretot-Richert G. Ducharme M. Monsarat-Chanon H.. (2021). Custom-fitted in- and around-the-ear sensors for unobtrusive and on-the-go EEG acquisitions: development and validation. Sensors 21:2953. doi: 10.3390/s21092953, PMID: 33922456 PMC8122839

[ref49] Wang Z. Shi N. Zhang Y. Zheng N. Li H. Jiao Y.. (2023). Conformal in-ear bioelectronics for visual and auditory brain-computer interfaces. Nat. Commun. 14:4213. doi: 10.1038/s41467-023-39814-6, PMID: 37452047 PMC10349124

[ref50] Xu Y. De La Paz E. Paul A. Mahato K. Sempionatto J. R. Tostado N.. (2023). In-ear integrated sensor Array for the continuous monitoring of brain activity and of lactate in sweat. Nat. Biomed. Eng. 7, 1307–1320. doi: 10.1038/s41551-023-01095-1, PMID: 37770754 PMC10589098

[ref51] Yarici M. C. Thornton M. Mandic D. P. (2023). Ear-EEG sensitivity modeling for neural sources and ocular artifacts. Front. Neurosci. 16:997377. doi: 10.3389/fnins.2022.997377, PMID: 36699519 PMC9868963

[ref52] Zibrandtsen I. C. Kidmose P. Christensen C. B. Kjaer T. W. (2017). Ear-EEG detects ictal and Interictal abnormalities in focal and generalized epilepsy – a comparison with scalp EEG monitoring. Clin. Neurophysiol. 128, 2454–2461. doi: 10.1016/j.clinph.2017.09.115 29096220

[ref53] Zibrandtsen I. Kidmose P. Otto M. Ibsen J. Kjaer T. W. (2016). Case comparison of sleep features from ear-EEG and scalp-EEG. Sleep Sci 9, 69–72. doi: 10.1016/j.slsci.2016.05.006 27656268 PMC5021956

